# Sex‐specific prey partitioning in breeding piscivorous birds examined via a novel, noninvasive approach

**DOI:** 10.1002/ece3.4421

**Published:** 2018-08-14

**Authors:** Bettina Thalinger, Johannes Oehm, Christiane Zeisler, Julia Vorhauser, Michael Traugott

**Affiliations:** ^1^ Institute of Ecology University of Innsbruck Innsbruck Austria

**Keywords:** CHD 1 gene, feeding ecology, fish‐length regression analysis, molecular prey detection, molecular sexing, piscivory

## Abstract

Piscivorous birds frequently display sex‐specific differences in their hunting and feeding behavior, which lead to diverging impacts on prey populations. Cormorants (Phalacrocoracidae), for example, were previously studied to examine dietary differences between the sexes and males were found to consume larger fish in coastal areas during autumn and winter. However, information on prey partitioning during breeding and generally on sex‐specific foraging in inland waters is missing. Here, we assess sex‐specific prey choice of Great Cormorants (*Phalacrocorax carbo*) during two subsequent breeding seasons in the Central European Alpine foreland, an area characterized by numerous stagnant and flowing waters in close proximity to each other. We developed a unique, noninvasive approach and applied it to regurgitated pellets: molecular cormorant sexing combined with molecular fish identification and fish‐length regression analysis performed on prey hard parts. Altogether, 364 pellets delivered information on both, bird sex, and consumed prey. The sexes differed significantly in their overall prey composition, even though *Perca fluviatilis*,* Rutilus rutilus*, and *Coregonus* spp. represented the main food source for both. Albeit prey composition did not indicate the use of different water bodies by the sexes, male diet was characterized by higher prey diversity within a pellet and the consumption of larger fish. The current findings show that female and male cormorants to some extent target the available prey spectrum at different levels. Finally, the comprehensive and noninvasive approach has great potential for application in studies of other piscivorous bird species.

## 1 INTRODUCTION

The ecology of a species can be strongly influenced by sex‐specific differences, and in birds, sexual dimorphism regarding appearance, size, and behavior is a widespread phenomenon (Slatkin, 1984). For seabirds, such variations in feeding and behavioral ecology have been frequently observed (e.g., Bearhop et al., 2006; Widmann et al., 2015), and they can strongly influence predation pressure exerted on prey populations and interfere with management and conservation issues (Phillips, McGill, Dawson, & Bearhop, 2011; Thalmann, Baker, Hindell, Double, & Gales, 2007). However, investigations on sex‐specific prey choice and potential resource partitioning of breeding piscivorous birds so far remain mostly limited to marine environments (e.g., Cleasby et al., 2015; Ismar, Raubenheimer, Bury, Millar, & Hauber, 2017; Robinson, Forbes, & Hebert, 2009).

Amongst other species, cormorants and shags (Phalacrocoracidae) are abundant generalist piscivores in marine and freshwater environments around the world (del Hoyo, Elliot, & Sargatal, 1992), and some differences between males and females have been studied, albeit not extensively. Whilst otherwise visually indistinguishable, these birds usually display a sexual size dimorphism: Males are generally larger than females and have 8%–19% higher body mass (Croxall, 1995; Fonteneau, Paillisson, & Marion, 2009; Koffijberg & vanEerden, 1995; Liordos & Goutner, 2008). In coastal environments, this further manifests in sex‐specific diving performance, with males commonly diving deeper (e.g., Gomez Laich, Quintana, Shepard, & Wilson, 2012; Watanuki, Kato, & Naito, 1996), the use of different foraging areas (Anderson, Roby, & Collis, 2004; Quintana, Wilson, Dell'Arciprete, Shepard, & Laich, 2011), and distinct response to environmental conditions such as strong winds (Lewis, Phillips, Burthe, Wanless, & Daunt, 2015).

**Figure 1:**
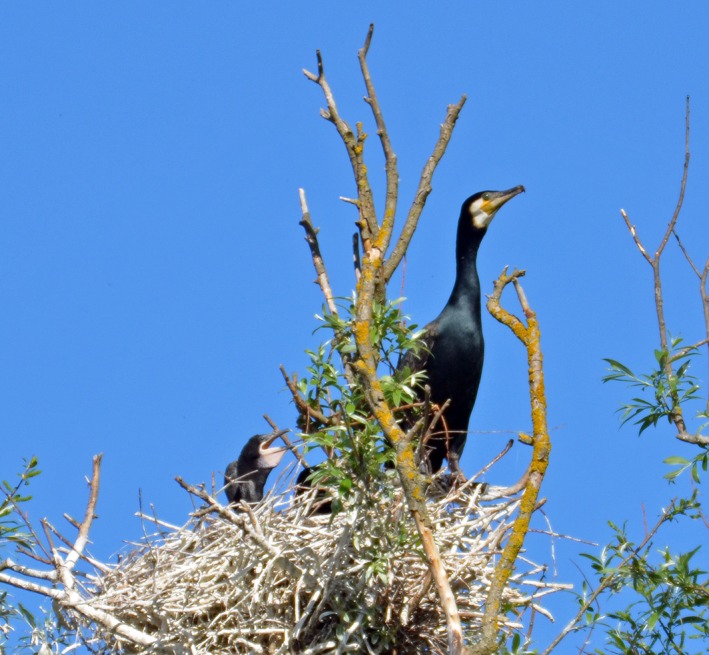
Adult Great Cormorant (*Phalacrocorax carbo*) and chicks at the colony located on the shore of Lake Chiemsee (Germany). © Michael Traugott [Colour figure can be viewed at http://wileyonlinelibrary.com]

In the Antarctic, the diet of male cormorants and shags, compared to females, has been characterized by prey from higher trophic levels (Bearhop et al., 2006) and larger fish leading to resource partitioning between the sexes (Casaux, Favero, Silva, & Baroni, 2001; Kato, Nishiumi, & Naito, 1996). For the Great Cormorant (*Phalacrocorax carbo*, further on “cormorant”; Figure 1), females were found to consume smaller fish than males based on stomach content analysis (Fonteneau et al., 2009; Koffijberg & vanEerden, 1995; Liordos & Goutner, 2009). But whilst cormorants in the Netherlands and France access the same prey spectrum (Fonteneau et al., 2009; Koffijberg & vanEerden, 1995), the sexes consume different fish species along the Greek coast (Liordos & Goutner, 2009). These studies have all been conducted in coastal lowlands and focused on the overwintering period of the birds whereas information on sex‐specific foraging in structurally diverse inland waters during the breeding season is missing.

The great variety of freshwater habitats in the Central European Alpine foreland with a diverse fish community in both stagnant and flowing waters provides cormorants with manifold hunting grounds in close vicinity to each other (Marzano, Carss, & Cheyne, 2013). These might be used differently by breeding females and males in terms of the fish species consumed and their size. In this region, the cormorant breeding season starts in early spring and can be divided into construction or renewal of the nest, 1 month of incubation, followed by chick‐provisioning until late summer. The chicks are fed via regurgitated stomach content, that is strongly digested and liquefied fish remains during early rearing and gradually switching to less digested and whole fish (Rutschke, 1998; Trauttmansdorff & Wassermann, 1995).

Every morning, adult cormorants usually regurgitate a pellet containing indigestible remains of the prey consumed on the previous day (Zijlstra & vanEerden, 1995). Juveniles start pellet regurgitation at the age of 2 months at irregular intervals (Trauttmansdorff & Wassermann, 1995). To obtain high sample numbers and avoid difficulties associated with aquiring permits for shooting or catching and handling of cormorants (protected by EU legislation under Directive 2009/147/EC (European Parliament & Council of the European Union 2009)), regurgitated pellets have been extensively used to study cormorant feeding ecology in European freshwaters (e.g., Dias, Morais, Leopold, Campos, & Antunes, 2012; Keller, 1998). The indigestible hard parts can be utilized to determine the species and number of the consumed fish (Barrett et al., 2007). Furthermore, specific hard parts such as otoliths and chewing pads can be employed to estimate fish length (e.g., Emmrich & Düttmann, 2011). Due to the high degradation of some fish remains during the digestion process and strong morphological similarities in hard part shape between some prey species, visual examination alone is not always sufficient for species‐specific prey identification (cp. Thalinger, Oehm, Mayr, Obwexer, Zeisler & Traugott, 2016). To bypass these shortcomings, molecular tools have been at the forefront of noninvasive approaches to study the feeding ecology of a wide range of vertebrate and invertebrate species (cp. King, Read, Traugott, & Symondson, 2008; Pompanon et al., 2012). Furthermore, molecular identification systems for Central European fish species have already been established and shown to be highly efficient when applied to cormorant pellets (Oehm, Thalinger, Eisenkölbl, & Traugott, 2017; Thalinger et al., 2016). The combination of both morphological (providing prey fish individual numbers and size) and molecular (providing the complete prey spectrum) methods enables the highest possible resolution for studies of cormorant prey choice.

Aside from indigestible fish hard parts and fish DNA, pellets contain mucus stemming from the bird's stomach wall (Rutschke, 1998), which in turn contains DNA of the pellet‐producing cormorant. This enables the application of molecular methods to determine the sex of the pellet‐producing bird. Regarding the use of consumer DNA, a variety of methods for sex determination (“molecular sexing”) and individual identification have been developed (e.g., Kohn & Wayne, 1997; Wultsch, Waits, & Kelly, 2014). For birds, molecular sexing has been commonly applied since the late 1990s to determine the sex of indistinguishable adult birds, juveniles, and embryos (Morinha et al., 2013). Generally, the technique is based on sequence variations of the sex chromosomes, and depending on the species under investigation, different primers have been developed and applied in allele‐specific PCRs (Dawson, Dos Remedios, & Horsburgh, 2016; Morinha, Cabral, & Bastos, 2012). For European cormorants, Thanou, Giokas, Goutner, Liordos, and Fraguedakis‐Tsolis (2013) found the primers 2550F and 2718R (Fridolfsson & Ellegren, 1999), targeting the chromodomain‐helicase‐DNA‐binding protein 1 gene (CHD1) to be best suited for molecular sexing. Traditionally, blood, feathers, muscle tissue, egg shells, and buccal swab samples have been used as a source of bird DNA (Griffiths, Daan, & Dijkstra, 1996; Morinha et al., 2013; Thanou et al., 2013), but recently, molecular sexing has also been successfully carried out with fecal samples (e.g., Faux, McInnes, & Jarman, 2014; Jarman et al., 2013).

Here, we apply molecular sexing for the first time to cormorant pellets in combination with fish‐length regression analysis of hard parts and molecular prey identification. Beyond that, new regression formulae were derived to improve fish‐length calculations for small fish and species occurring in Alpine foreland freshwaters. Based on the results obtained from other cormorant species, we hypothesized the following: (H1) Prey diversity and composition differ between females and males, as the larger males can potentially access a broader spectrum of fish. Depending on the consumed species, this could also indicate the use of different foraging grounds; (H2) male cormorants feed on longer fish than females; and (H3) female cormorants consume significantly more fish individuals than males to compensate for the inaccessible larger prey individuals.

## 2 METHODS

### 2.1 Pellet collection, lysis, and DNA extraction

During the breeding seasons (March–August) 2012 and 2013, 633 freshly regurgitated cormorant pellets, produced during the respective sampling mornings, were collected every second week at the shore of Chiemsee (Bavaria, Germany; see Supporting Information SI 1 for sampling dates). In 2012, sampling took place at one cormorant breeding colony in the estuary of the River Tiroler Achen (N 47.862839, E 12.503541). In 2013, part of the cormorants established a subcolony (N 47.859971, E 12.509115) located ~1 km from the original spot and pellets were collected at both places. In general, sampling was carried out on two consecutive days. On the first day, all pellets, which had been produced since the last sampling event, were individually collected using everted plastic bags. On the following day, all fresh pellets were collected in the same way. The herewith‐presented work is solely based on fresh pellets (not older than 24 hr) collected on the second day, which were transported in a cooling box to the laboratory where they were stored at −32°C until further analysis.

DNA extraction from pellets was carried out as described in Thalinger et al. (2016). In brief, pellets were defrosted and lysed using TES‐buffer and Proteinase K and incubated for at least 6 hr (lysis starting in the morning) and a maximum of 12 hr (lysis starting in the evening) at 56°C. Then, 1.5 ml of each pellet lysate was transferred into a new reaction tube whilst subjecting the remaining sample to morphological prey identification (see below). DNA extraction was carried out using the BioSprint 96 DNA Blood Kit (QIAGEN, Hilden, Germany) on the BioSprint 96 instrument; program “BS96 Tissue” (QIAGEN) in accordance with the manufacturer's instructions, except for elution, which was carried out in 1× TE‐buffer instead of AE‐buffer. Four extraction negative controls were included in every batch of 96 samples, which were later checked for cross‐contamination (see below).

### 2.2 Molecular sexing

The primers 2550F and 2718R, targeting the CHD1 gene (Fridolfsson & Ellegren, 1999) and resulting in a single amplicon (652 bp) for male cormorants and two amplicons (459 and 652 bp) for female cormorants, were used for molecular sexing (see Supporting Information SI 2 for PCR optimization, final PCR conditions, and electrophoretic visualization). To confirm the applicability of this approach, DNA amplicons stemming from male muscle tissue (*n* = 3) and pellets (*n* = 8) were sequenced and female‐specific amplicons generated from muscle tissue (*n* = 3) and pellets samples (*n* = 8) were cleaned up using the QIAquick Gel Extraction Kit (QIAGEN) and subsequently sequenced. All of the generated sequences could be identified as cormorant‐specific CHD1Z or CHD1W, respectively (Supporting Information SI 3). During capillary electrophoresis, it was noted that the female‐specific amplicon (459 bp) was often weaker than the amplicon produced by both sexes (652 bp). Consequently, a pellet containing a small amount of amplifiable DNA could result in only a weak signal at 652 bp and be erroneously classified as produced by a male cormorant. Hence, the threshold for pellets producing a single amplicon at 652 bp was set at 0.3 relative fluorescence units (RFUs; thrice as high as for signals at 459 bp). The 22 pellets producing a single 652‐bp amplicon with less than 0.3 RFUs were excluded from further analyses.

### 2.3 Molecular prey identification

Pellet DNA extracts, which could be successfully sexed, were subjected to a two‐step multiplex PCR system reliably detecting and identifying fish species occurring in the study area from as little as 25 double strands of mitochondrial target DNA (for a detailed description, see Thalinger et al., 2016). The system is comprised of six multiplex PCR assays, in a first step permitting fish identification on a family‐specific level (“FishTax” assay), and in a second step enabling species‐specific identification of Salmoniformes, Percomorphaceae, and Cypriniformes via the assays “SalForm,” “PercMorph,” “CypForm 1‐3.” This two‐step multiplex PCR system has been previously used to successfully identify fish DNA from cormorant pellets (Oehm et al., 2017; Thalinger et al., 2016. Amplifications were carried out with the Multiplex PCR Kit (QIAGEN) in 10 μl PCRs. Per reaction, 1.5 μl (FishTax) or 3.2 μl (all other assays) of DNA extract, one‐time reaction mix, 5 μg BSA, 30 mM TMAC, primers in respective concentrations and PCR‐grade water (FishTax assay only) were combined and subjected to thermocycling at optimized conditions: 15 min at 95°C, 35 cycles of 30 s at 94°C, 90 s at 64°C (FishTax, SalForm, PercMorph, CypForm 2) or 66°C (CypForm 1, CypForm 3), 1 min at 72°C, and 10 min at 72°C once (Thalinger et al., 2016). In capillary electrophoresis (Supporting Information SI 2), all target bands with RFU ≥0.1 were counted as positive. All extraction negative controls were checked with the FishTax assay and resulted negative, as well as all PCR‐negative controls included in each individual PCR.

### 2.4 Morphological prey identification

Pellets were sieved (0.5 mm mesh size) and hard parts suitable for fish identification (otoliths, pharyngeal bones, chewing pads, and jaws) were selected. Fish prey remains were identified using the following identification keys: Härkönen (1986), Knollseisen (1996), Veldkamp (1995b) as well as reference collections provided by Werner Suter (Swiss Federal Research Institute, Birmensdorf, Switzerland), Josef Trauttmansdorff (Otto König Institute, Stockerau, Austria), and the Bavarian State Collection of Zoology (Munich, Germany). To obtain fish‐length regression formulae for Alpine foreland fish, reference fish samples of 13 species (*n* = 298) caught in waters around Chiemsee between August 2013 and March 2014 were used. The total fish length was measured before obtaining otoliths, pharyngeal bones, and chewing pads. The length/width of the hard parts was determined to the nearest 0.1 mm to establish regression formulae for fish‐length calculations (Supporting Information SI 4). Fish‐length regression analysis was applied to those sagittal otoliths, pharyngeal bones, dentaries, and chewing pads, which were obtained from cormorant pellets, could be species‐specifically identified, and showed no signs of digestive wear. Hard parts were measured as described above, and for all fish species, the formula with the best fit at the respective fish length was selected amongst previously published work and the newly generated regression formulae. Altogether, 20 self‐established regression formulae for 13 fish species supported by 10 literature‐based formulae were used for calculations of total fish length (Supporting Information SI 4).

Fish individual numbers were estimated through counts of eye lenses, otoliths, and chewing pads. Pairwise occurring hard parts were sorted per pellet and fish species, and only one measurement was made per pair, resulting in one value per individual. If a pairwise occurring hard part could not be matched, it was measured and counted as one individual. Chewing pads, dentaries, and pharyngeal bones were also matched per species and size. Due to the lack of a regression formula, seven chewing pads and two pharyngeal bones of Danube bleak (*Alburnus mento*) detected in two pellets had to be excluded from further analyses.

### 2.5 Statistical analysis

All calculations and visualizations were carried out in R (R Development Core Team 2017) using the packages “vegan” (Oksanen et al., 2016), “ggplot2” (Wickham, 2009), “gridExtra” (Auguie, 2015), and “ggpubr” (Kassambara, 2017).

A chi‐square‐test was carried out to test whether the sex ratio differed significantly between pellets containing measurable fish remains and those without such. The proportion of each fish species in total detections was graphed for three datasets: (a) morphological detections based on counts of measurable fish, (b) morphological detections as presence/absence data, and (c) molecular detections (presence/absence data). Per dataset, the proportions were calculated separately for the two sexes, the two breeding seasons combined, and each of the two breeding seasons separately. The datasets were visually examined for differences in prey spectrum between pellets produced by adult cormorants during the first 3 months of sampling and pellets produced by adults and juveniles at later sampling events. No distinct difference in the prey spectrum was observed and detection rates differed on average 2.2% (molecular detections) and 2.5% (count‐based morphological detections), thus pellets from later sampling events were included in all further analyses.

For female‐ and male‐produced pellets, multivariate homogeneity of groups dispersions, that is β‐diversity (PERMDISP; 9,999 permutations; vegan function “betadisper”) and permutational multivariate analysis of variance, that is differences in composition and relative abundances of fish species (permANOVA; 9,999 permutations; vegan function “adonis”) were tested (Anderson, 2001, 2006; Anderson, Ellingsen, & McArdle, 2006). The analyses were carried out for the three datasets (a, b, c) described above and per dataset repeated for two breeding seasons combined and each of the two breeding seasons separately. The Morisita–Horn metric (Horn, 1966) was used for count data as it passes all quality criteria for abundance‐based β‐diversity indices (Barwell, Isaac, & Kunin, 2015) and is robust concerning undersampling (Beck, Holloway, & Schwanghart, 2013). For presence/absence data, the probabilistic Raup–Crick dissimilarity metric was selected as the investigated samples can be considered to be taken from the same regional species pool (Chase, Kraft, Smith, Vellend, & Inouye, 2011).

For comparisons of mean overall fish length and number of consumed individuals between the sexes, Mann–Whitney–Wilcoxon tests were used. To reduce the effect of outliers, that is few pellets with extremely high individual numbers, mean fish length per species was calculated for each pellet and used for all further calculations. A Kruskal–Wallis test was applied to test for significant differences in mean fish length between the fish species found in individual pellets. To examine sex‐specific differences in fish length for each species separately, and to compare the mean fish length per pellet of the two most abundant prey species (perch *Perca fluviatilis* and roach *Rutilus rutilus*) between the sexes, Mann–Whitney–Wilcoxon tests with Holm–Sidak corrected *p*‐values were calculated.

Based on the morphological fish‐count dataset, a canonical correspondence analysis (CCA) was calculated to assess the influence of constraining variables on prey species composition in female and male diets: Per pellet, the number of measurable fish per species was square‐root‐transformed and used as community matrix. The sex of the pellet‐producing cormorant, the number of measurable fish individuals in the pellet, and the mean fish length per pellet were entered as constraints. CCAs were calculated for the total dataset and the breeding seasons 2012 and 2013 separately. A permANOVA (9,999 permutations) was carried out to test for significant effects of the constraining variables.

## 3 RESULTS

Of the 633 collected pellets, 415 could be reliably assigned to one of the two sexes with 45% classified as “produced by a female cormorant” (further on “female”) and 55% as “produced by a male cormorant” (further on “male”). Of the sexed pellets, 354 delivered molecular information on consumed fish (Supporting Information SI 1) and 313 contained fish hard parts of 1,572 fish individuals. However, only 194 of these pellets (47% female, 53% male) contained hard parts identifiable to species level and suitable for fish‐length regression analysis (*n* = 1,180; 75%; 14 species; Supporting Information SI 1). The female / male ratio of the 221 pellets without measurable fish (43% female; 57% male) was not significantly different from the female / male ratio of pellets containing such hard parts (χ^2^ = 0.95; *p* = 0.28).

**Figure 2:**
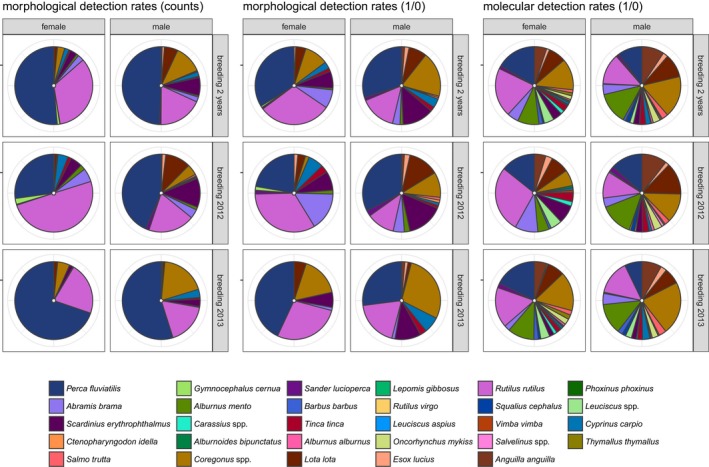
The detection rates of prey fish based on counts and presence/absence data. The left block is based on counts of measurable fish from cormorant pellets, the middle block is based on presence/absence data obtained from these measurable fish, and the right block is based on molecular data (presence/absence). Per block, the proportion of each fish species in total counts/detections is displayed separately for the two sexes (female and male) and the total dataset, breeding season 2012, and breeding season 2013 [Colour figure can be viewed at http://wileyonlinelibrary.com]

Based on measurable fish hard parts, 12 of the 14 species (listed in Figure 2) were detected in both female and male pellets: Grass carp (*Ctenopharyngodon idella*,* n* = 1), brown trout (*Salmo trutta*,* n* = 2), and eel (*Anguilla anguilla*,* n* = 2) were only found in male pellets; ruffe (*Gymnocephalus cernua*,* n* = 7) only in female pellets. Perch was the most frequently detected fish species in the hard part analysis (female: 46%; male: 43%; Figure 2). From 2012 to 2013, the proportion of measurable perch and roach individuals in female diet changed adversely: perch increased 37% whilst roach decreased 26%. Furthermore, the proportion of whitefish (*Coregonus* spp.) increased from 4% to 17% in male pellets (Figure 2). Regarding molecular prey identification, 21 of 27 detected fish taxa (listed in Figure 2) occurred in both female and male pellets: Common sunfish (*Lepomis gibbosus*, 5%), minnow (*Phoxinus phoxinus*, 5%), cactus roach (*Rutilus virgo*, 5%), and char (*Salvelinus* spp., 9%) were detected only in male pellets; asp (*Leuciscus aspius*, 6%) and vimba (*Vimba vimba*, 6%) only in female pellets. Here, the most frequently detected taxa were roach for females (35%) and whitefish for males (30%; Figure 2).

From both morphological count‐based and morphological presence/absence data, pellets produced by female and male cormorants differed significantly in β‐diversity (mean distance to centroid smaller for female) in the total dataset, as well as in breeding season 2013 (Table 1). For molecular prey detection, such a significant difference was only detected in the breeding season 2012 (Table 1). Fish species composition differed significantly between the sexes for both morphological and molecular datasets (two breeding seasons combined and separated; Table 1). However, only for morphological presence/absence data obtained from 2012, and molecular data obtained from the total dataset and 2013, these results can be interpreted as true differences in prey species composition between the sexes, because no significant difference in β‐diversity (a fundamental assumption of permANOVA) was observed.

**Table 1:** Differences in β‐diversity (PERMDISP; 9,999 permutations) and composition/relative abundances of fish species (permANOVA; 9,999 permutations) between female and male cormorant pellets

Cormorant sex	Morphological count‐based data					Morphological presence/absence data					Molecular presence/absence data				
	permANOVA		PERMDISP			permANOVA		PERMDISP			permANOVA		PERMDISP		
	*R* ^2^	*p*‐value	*p*‐value	Mean distance to centroid		*R* ^2^	*p*‐value	*p*‐value	Mean distance to centroid		*R* ^2^	*p*‐value	*p*‐value	Mean distance to centroid	
				Female	Male				Female	Male				Female	Male
Breeding 2 years	0.024	0.001	**<0.001**	0.513	0.578	0.027	0.001	**<0.001**	0.459	0.539	0.022	**0.001**	0.193	0.542	0.565
Breeding 2012	0.078	0.001	**0.022**	0.506	0.566	0.086	**0.001**	0.09	0.471	0.524	0.071	0.001	**0.001**	0.453	0.562
Breeding 2013	0.036	0.001	**0.001**	0.474	0.557	0.038	0.001	**0.01**	0.448	0.521	0.019	**0.006**	0.941	0.557	0.559

*Note*

Analyses were carried out for morphological count‐based data, morphological presence/absence data and molecular data (presence/absence) for the total dataset and the two breeding seasons separately. Additionally, the mean distance to centroid after PERMDISP analysis is displayed for each dataset. Significant *p*‐values <0.05 are in bold, but for permANOVA only if PERMDISP does not lead to a significant difference between the sexes.

**Figure 3:**
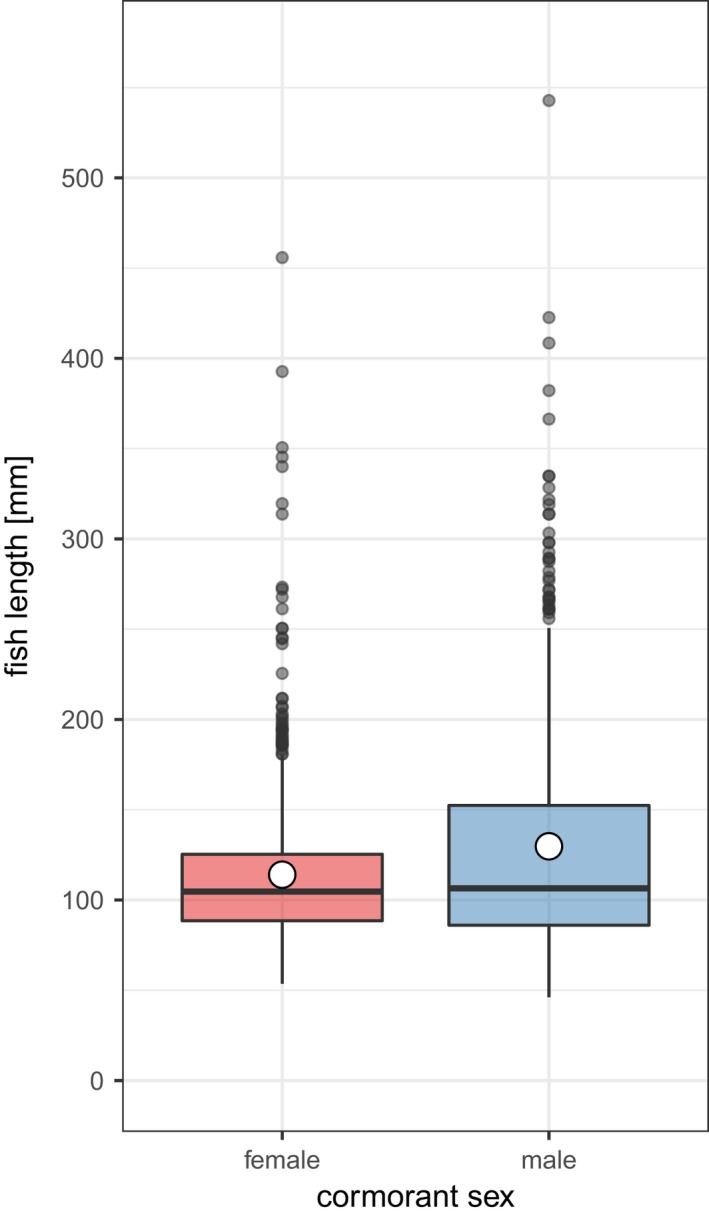
Estimated length of all measurable fish individuals found in cormorant pellets produced by females (651 fish) and males (529 fish). The white dot depicts the mean fish length per cormorant sex; the line the median. The fish consumed by females were significantly smaller compared to the ones eaten by males (female mean = 114 mm ± 44 mm *SD*; male mean = 130 mm ± 66 mm *SD*;* W* = 159,660, *p* = 0.03) [Colour figure can be viewed at http://wileyonlinelibrary.com]

Fish specimens stemming from female pellets (*n* = 651) had a mean length of 114 mm ± 44 mm *SD*, which was significantly smaller (*W* = 159,660; *p* = 0.03) than the fish found in male pellets (mean: 130 mm ± 66 mm *SD*;* n* = 529; Figure 3). Most pellets (67%) contained the remains of one to four measurable fish individuals, but six pellet samples contained over 35. No significant difference in the number of measurable fish per pellet could be detected between the two cormorant sexes (*W* = 22,003; *p* = 0.38; female: 3.51 ± 9.89 *SD*; male: 2.36 ± 5.51 *SD*).

**Figure 4:**
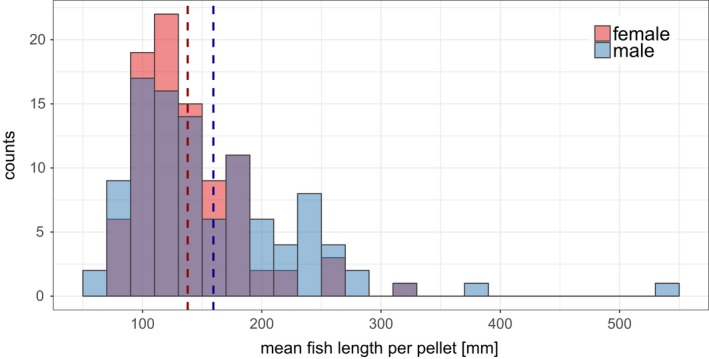
Frequency distribution of the mean fish length per pellet [mm] independent of fish species for both cormorant sexes. Mean fish lengths are grouped per 20 mm, and the dashed lines mark the mean per sex (red: female: 138 mm ± 45 mm *SD*; blue: male: 159 mm ± 74 mm *SD*;* W* = 4436.5, *p* = 0.09) [Colour figure can be viewed at http://wileyonlinelibrary.com]

**Figure 5:**
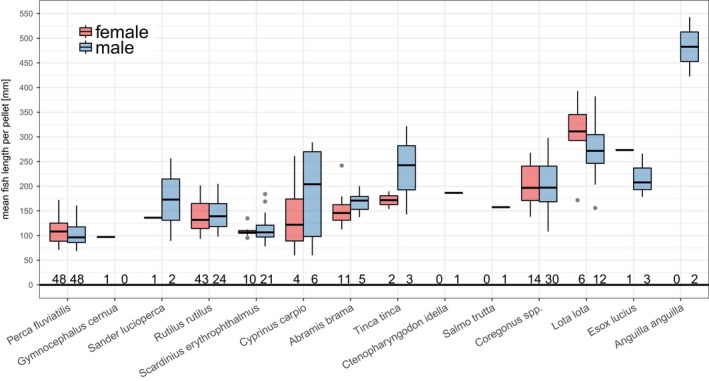
Mean fish length [mm] per pellet separately displayed for female and male‐produced cormorant pellets and each fish species for which a length regression formula was available. Numbers on the *x*‐axis display per cormorant sex the pellets in which the respective fish species was detected. The horizontal line depicts the median; for none of the fish species, significant differences in mean per pellet fish length were found between the sexes [Colour figure can be viewed at http://wileyonlinelibrary.com]

The mean fish length per pellet was greater in males (mean: 159 mm ± 74 mm *SD*) than in females (mean: 138 mm ± 45 mm; *W* = 3,931.5; *p* = 0.09), and the associated frequency distribution was right‐skewed for males compared to females (Figure 4). Differences in mean fish lengths were significant between fish species (χ^2^ = 185.9, *p* < 0.0001), but not between sexes per fish species (Figure 5). Furthermore, the mean perch and roach length per pellet did not differ between the sexes in any of the two breeding seasons (perch: 2012: *W* = 224.5; *p* = 0.45; 2013: *W* = 383; *p* = 0.45; roach: 2012: *W* = 97; *p* = 0.75; 2013: *W* = 128; *p* = 0.41).

**Figure 6:**
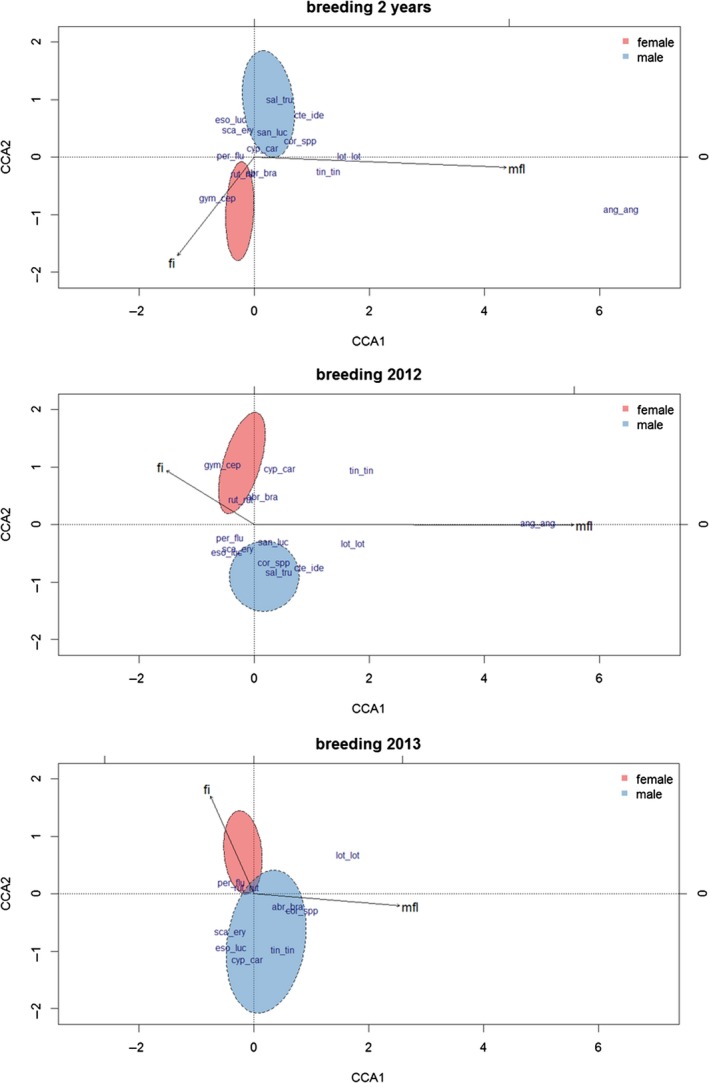
Canonical correspondence analyses (CCAs) calculated for the two breeding seasons (194 pellets), and the breeding seasons 2012 (90 pellets) and 2013 (104 pellets) separately. The ellipsoids depict the 95% confidence region around the centroids of female‐ and male‐produced pellets. For eigenvalues and percentage of variance explained by the CCA axes, see Table 3. Constraints and fish species are abbreviated as follows: fi: number of measurable fish individuals per pellet, mfl: mean fish length per pellet, abr_bra: *Abramis brama*, ang_ang: *Anguilla anguilla*, cor_spp: *Coregonus* spp., cte_ide: *Ctenopharyngodon idella*, cyp_car: *Cyprinus carpio*, eso_luc: *Esox lucius*, gym_cer: *Gymnocephalus cernua*, lot_lot: *Lota lota*, per_flu: *Perca fluviatilis*, rut_rut: *Rutilus rutilus*, san_luc: *Sander lucioperca*, sca_ery: *Scardinius erythrophthalmus*, tin_tin: *Tinca tinca* [Colour figure can be viewed at http://wileyonlinelibrary.com]

Cormorant sex and mean fish length per pellet were highly significant constraints in all CCAs (*p* < 0.005; Table 2) with nonoverlapping confidence areas between the two sexes for the total dataset and the breeding season 2012 (Figure 6). Contrastingly, the number of measurable fish individuals had no significant constraining effect (Table 2). In the total dataset, the female pellets clustered toward higher fish individual numbers per pellet. Female pellets were in both years characterized by roach; male pellets by a more diverse set of fish species such as pike (*Esox lucius*), pike‐perch (*Sander lucioperca*), rudd (*Scardinius erythrophthalmus*) and whitefish; this difference was more pronounced in the breeding season 2012 than 2013 (Table 3; Figure 6).

**Table 2:** Results of permutational ANOVAs calculated for each of the three CCAs (two breeding seasons combined, breeding season 2012 and 2013) with 9,999 permutations; significant p‐values <0.05 are in bold

permANOVA (9,999 permutations)				
	Constraints	χ^2^	*F*	*p*‐value
Breeding 2 years	Cormorant sex	0.09	2.64	**<0.0001**
*R* ^2^ = 0.08	Number of measurable fish per pellet	0.05	1.59	0.1484
	Mean fish length per pellet	0.43	12.81	**<0.0001**
Breeding 2012	Cormorant sex	0.21	3.12	**<0.0001**
*R* ^2^ = 0.12	Number of measurable fish per pellet	0.07	1.00	0.3274
	Mean fish length per pellet	0.46	6.80	**<0.0001**
Breeding 2013	Cormorant sex	0.11	2.33	**0.0018**
*R* ^2^ = 0.13	Number of measurable fish per pellet	0.09	1.98	0.0905
	Mean fish length per pellet	0.48	10.59	**<0.0001**

*Note*

Per CCA the *R* ^2^, and per constraint, the chi‐square, *F*‐statistic, and *p*‐value are provided.

**Table 3:** Eigenvalues and the proportion of variance explained for the two CCA axes displayed in Figure 6

CCA summary	CCA1	CCA2
Breeding 2 years		
Eigenvalue	0.48	0.06
Proportion of variance explained	0.07	0.01
Breeding 2012		
Eigenvalue	0.49	0.20
Proportion of variance explained	0.08	0.03
Breeding 2013		
Eigenvalue	0.52	0.13
Proportion of variance explained	0.10	0.02

*Note*

The values are provided for the two breeding seasons combined, breeding season 2012 and 2013 separately.

## 4 DISCUSSION

Our study is the first to describe the successful application of molecular sexing to regurgitated pellets of piscivorous birds in combination with molecular prey identification and morphological prey hard part analysis, maximizing the dietary information obtainable per sample. This innovative approach allowed unravelling sex‐specific patterns of prey choice in cormorants in a noninvasive way and is easily adaptable to other piscivorous birds. As hypothesized (H1), we found conclusive evidence for sex‐specific prey partitioning in our 2‐year dataset with females consuming a less diverse set of fish species compared to males. Females and males also differed significantly in their overall prey composition, even though morphological analysis found perch and roach to be the most frequently consumed species for both sexes. Based on all measurable fish individuals, male cormorants fed on longer fish than females (H2). However, there were no significant differences in fish length between the two sexes for individual species. Thus, the difference in fish size between the sexes seems to be driven by general distinctions in dietary choice and sparsely consumed fish species. Our data also go in the direction of supporting the third hypothesis proposing that the average meal of females contains more fish individuals, albeit this difference was not significant.

The daily diet of male cormorants was characterized by a more diverse prey spectrum. However, this difference in prey diversity per pellet was not uniform between datasets derived by either morphological or molecular prey identification. In the total dataset, males exhibited a significantly broader prey diversity per pellet regarding morphological prey identification, whereas such a difference was not observed with the molecularly derived data. For a standardized fish intake, cormorant sex has not been found to affect prey detection in dietary samples (Thalinger et al., 2017). Hence, the lower taxonomic resolution of hard part analysis is likely to cause this difference (McKay, Robinson, Carss, & Parrott, 2003; Oehm et al., 2017). The examination of individual breeding seasons confirmed this pattern for 2013, but in 2012, a significant difference in prey diversity per pellet occurred in the molecular data set, caused by nine rarely occurring fish species, which were not molecularly detected in female pellets. For sex‐specific examinations of both prey diversity per pellet and the total prey spectrum, the applied molecular approach seems to be more reliable as it has a higher taxonomic resolution than morphological analysis and the individual primer pairs are known to be species‐specific and standardized concerning amplification success (Thalinger et al., 2016).

The overall greater fish length in male‐produced pellets indicates a general difference in accessible fish length between the sexes related to the species’ size dimorphism with males having 8%–19% increased mass compared to females (Fonteneau et al., 2009; Koffijberg & vanEerden, 1995; Liordos & Goutner, 2008). However, sex‐specific hunting strategies could also cause this pattern. So far, two hunting strategies, social and solitary fishing, have been described for cormorants (Carss, 2003; Gremillet, Argentin, Schulte, & Culik, 1998; Liordos & Goutner, 2009; Rutschke, 1998). The high numbers and small size of the predominantly consumed schooling fish, perch and cyprinids, indicate that in the present study, cormorants were hunting in groups in shallow waters (Cosolo, Ferrero, & Sponza, 2010). The large prey fish segregating the diets of males from females suggest that males to some extent evade food competition via solitary hunting in deeper waters (Rutschke, 1998). However, no size difference was detectable for individual species: Frequently detected fish (perch and roach) were consumed in the same size range, but low numbers of measurable fish hard parts hindered the detection of significant differences for other species. Nevertheless, the combination of these sparsely consumed fish potentially has a strong impact on the significant overall difference in fish length between the sexes.

The molecularly derived species‐specific prey identification can also be used to assess whether males and females use distinct foraging habitats, that is stagnant and flowing waters represented by typically lacustrine (e.g., whitefish) and riverine species (e.g., brown trout *Salmo trutta*), respectively. Four riverine salmonid species occurred in the present study, and detection rates were higher in male pellets for all of them. This could be indicative of habitat partitioning between the sexes (Anderson et al., 2004; Quintana et al., 2011). However, riverine species accounted for less than 5% of the total detections per year and with two exceptions (12 July 2012: 38%; 13 August 2013: 50%) never occurred in more than 18% of the pellets analyzed per sampling event. Thus, it is possible that only few male individuals showed preferences for riverine foraging grounds whilst the majority of cormorants foraged preferentially at Chiemsee or other stagnant waters close to the breeding site.

The distinct partitioning of fish resources to reduce intersexual competition as found in marine habitats (Casaux & Barrera‐Oro, 2006; Liordos & Goutner, 2009) seems to be less relevant in the Alpine foreland: both sexes most frequently consumed perch, roach, and whitefish albeit in different proportions. However, whitefish detections were higher in male pellets (molecular: 30%; morphological: 10%) than female pellets (molecular: 23%; morphological: 2%). This difference could be caused by an interaction of diel vertical whitefish movements and sex‐specific daily foraging patterns in cormorants. On the one hand, whitefish are predominantly planktivorous, reside at greater depths during the day, and feed close to the lake surface at night (Mehner, 2012). On the other hand, sex‐specific foraging patterns with females hunting from morning to noon and males on the second half of the day have been observed for King Cormorants, Japanese Cormorants (*Phalacrocorax capillatus*), and Great Cormorants (Kato, Watanuki, Shaughnessy, Le Maho, & Naito, 1999; Kato et al., 2000; Platteeuw & vanEerden, 1995). It seems plausible that males hunting in the late afternoon when whitefish already commence their ascent are likely to catch this species at Chiemsee. Furthermore, males diving deeper than females have been reported for other cormorant species (e.g., Gomez Laich et al., 2012; Watanuki et al., 1996); this could make whitefish in the Alpine foreland even better accessible for males.

During the first 3 months of each breeding season, pellets were produced by adult cormorants. For later samplings, it is possible that the collected pellets contained the fish fed to nestlings or fish consumed by adults for self‐provisioning. Molecular sexing most likely reveals the sex of the pellet‐producing cormorant and not the sex of adults feeding chicks as pellets are encased in stomach mucus of the pellet‐producing bird and usually produced after overnight digestion (Rutschke, 1998; Zijlstra & vanEerden, 1995). Epithelial cells passed on from adult to chick are thus unlikely to withstand this process. Even though, samples obtained during early breeding season did on average hardly differ from later on obtained samples (2.2% (molecular detections) and 2.5% (count‐based morphological detections)), nestlings could potentially bias the results of the present study. But, young cormorants completely digest their fish meals due to their high calcium needs (Zijlstra & vanEerden, 1995) and after starting pellet production at 2 months age, regurgitate pellets very irregularly for the following 2–3 months until fledging (Trauttmansdorff & Wassermann, 1995). As only 35% of the analyzed pellets were collected during the time of irregular pellet production by nestlings, the effect of chick‐produced pellets on the current dataset should be minute. Differences in diet between chick‐provisioning and self‐provisioning, as found for some seabirds (Fijn, Van Franeker, & Trathan, 2012; Wilson, Daunt, & Wanless, 2004), could also potentially affect the herewith‐presented results. However, such variations are mostly associated with single prey loading species and a limited number of possible foraging trips (e.g., Wilson et al., 2004). For cormorants, highly efficient generalist hunters (Gremillet et al., 2004) feeding their chicks via regurgitated stomach content (Rutschke, 1998), both factors are nonrestricting. Hence, dietary differences between chick‐provisioning and self‐provisioning are unlikely.

Generally, female cormorants consumed significantly smaller fish than males and their pellets contained remains of more prey individuals, but it cannot be stated that females compensate for smaller prey size by higher numbers of prey items, as the latter difference was not significant. These results fit to data obtained from forced regurgitations of King Cormorants (*Phalacrocorax albiventer*) (Kato et al., 1996), as well as cormorant stomach samples (Fonteneau et al., 2009; Koffijberg & vanEerden, 1995). The lack of a significant difference in prey number could also be caused by distinct energy requirements, which are lower for the smaller females (Gremillet, Storch, & Peters, 2000). Hence, it might not be necessary for female cormorants to compensate for smaller prey fish by consuming more individuals. Contrastingly, energy requirements during the egg‐laying period are similar between the sexes and incubation as well as chick rearing is more demanding for females (Gremillet, Schmid, & Culik, 1995; Platteeuw & vanEerden, 1995). Thus, the absence of significant differences regarding consumed fish individuals could also be due to the complete digestion of small fish preferably targeted by females (Johnstone, Harris, Wanless, & Graves, 1990; McKay et al., 2003).

The complete digestion of small fish individuals as well as small meals furthermore affects both molecular and morphological prey detection: In 15% of the pellets, molecular prey identification did not detect fish species, 22% of the cormorant pellets did not contain any prey hard parts at all compared to 6%–32% in previous hard‐part‐based studies (Dias et al., 2012; Keller, 1998), and 53% did not contain measurable hard parts. However, the fraction of pellets without measurable hard parts did not differ between the sexes. Other hard‐part‐based studies advise excluding remains with visible digestive wear from fish‐length regression analysis (Suter & Morel, 1996) or introduce correction factors for affected hard parts (Boström, Östman, Bergenius, & Lunneryd, 2012; Veldkamp, 1995a). We excluded all fish hard parts showing digestive wear as the molecular approach provided dietary information for 78% of the pellets without measurable fish remains. Nevertheless, two factors could still potentially affect the calculated fish lengths: First, the relation between fish growth and otolith growth differs depending on fish life stage, growth rate and environmental factors (e.g., Humston, Moore, Wass, Dennis, & Doss, 2015; Mugiya & Tanaka, 1992) eventually leading to inaccurate results of fish‐length regression analysis. Second, it has been previously found that fish length calculated from hard parts without visible digestive wear still tends to underestimate the size of the actually consumed fish for both sexes (reviewed by McKay et al., 2003; Ross, Johnson, & Adams, 2005). As all pellets are potentially affected by such inaccuracies, the detected differences in prey size and prey composition between female and male cormorants in the Alpine foreland are unlikely to be biased.

Whilst the majority of pellets collected could be reliably sexed, 160 pellets did not produce a result in PCR and 22 samples, initially classified as male pellets, were excluded from the analysis. This high number of inconclusive samples could be caused by different reasons: Whilst buccal swabs are successfully used for molecular sexing (e.g., Adam, Scharff, & Honarmand, 2014; Handel, Pajot, Talbot, & Sage, 2006), the reliability of pellets as a source for bird DNA remains to be further assessed. The DNA fragments amplified with the selected primers are comparably long (459 and 652 bp) for samples exposed to digestive processes (King et al., 2008). For molecular sexing of avian feces and degraded tissue materials, considerably shorter fragments (<300 bp) and different priming sites have been targeted to work around this issue (Dawson, Brekke, Dos Remedios, & Horsburgh, 2015; Dawson et al., 2016; Faux et al., 2014). Nevertheless, we chose to work with longer fragments in the present study as the primers have been previously found to be suitable for European cormorants (Thanou et al., 2013) and cormorant DNA should be contained in intact stomach lining cells. Additionally, detrimental environmental effects on samples such as UV‐radiation and rain (cp. Oehm, Juen, Nagiller, Neuhauser, & Traugott, 2011) were kept minimal by sampling pellets within a few hours after their production. In comparison with studies using the same primers for pure bird samples (Fridolfsson & Ellegren, 1999; Thanou et al., 2013), the herewith applied PCR conditions were more selective (i.e., annealing temperature of 55°C compared to 48–50°C) to avoid the amplification of fish DNA. Also, the presence of inhibitory substances (King et al., 2008) cannot be ruled out entirely even though cormorant pellets were successfully used for molecular identification of consumed fish. Finally, the inferior amplification success of the female‐specific DNA fragment leads to the exclusion of pellets, which were either correctly or incorrectly classified as males. Albeit the above factors did not always permit to determine the sex of the pellet‐producing cormorants, the present study shows the great potential of molecular sexing when applied to regurgitations. The protocol could be easily adapted for other pellet‐producing bird species and applied independent of dietary analyses.

## 5 CONCLUSIONS

Our findings demonstrate that Great Cormorants, the most abundant piscivorous birds along European inland freshwaters, exhibit sex‐specific prey partitioning during the breeding season. Whilst both sexes strongly rely on the same fish species, their overall prey composition was significantly different, the daily diet of females was less diverse compared to males, and males devoured on average larger fish individuals. The majority of fish species consumed by females and males inhabit lacustrine habitats; hence, a sex‐specific partitioning between foraging grounds cannot be supported by the current findings. Hard part analysis and molecular prey detection provided complementary results, the former on prey size and individual numbers, and the latter on the complete prey spectrum, thus enabling a comprehensive analysis of dietary differences. Finally, the novel combination of molecular sexing, molecular prey identification, and fish‐length regression analysis from the very same pellet presents a powerful, noninvasive tool for future investigations on the trophic ecology of protected and rare species.

## CONFLICT O FINTEREST

None declared.

## AUTHOR CONTRIBUTION

BT, JO, and MT conceived and designed the study. BT, JO, CZ, and field assistants carried out the field sampling. All molecular work was done by BT, JV and CZ, and morphological analysis was carried out by JO and JV. JO developed the regression formulae. BT analyzed the data obtained from cormorant pellets, compiled tables and figures, and wrote the manuscript, which was revised and improved by JO, CZ, and MT.

## DATA ACCESSIBILITY

The morphologically and molecularly obtained results regarding fish identity, fish length, and cormorant sex as well as the obtained CHD1Z and CHD1W sequences have been archived in the Dryad Digital Repository: https://doi.org/10.5061/dryad.b30c555.
