# Supplementary material for: Sex‐specific prey partitioning in breeding piscivorous birds examined via a novel, noninvasive approach
Source: Ecol Evol. 2018 Aug 14;8(17):8985–98. doi: 10.1002/ece3.4421 (PMC6157673; doi:10.1002/ece3.4421)
Supplement: Supplementary file 1 [file ECE3-8-8985-s001.pdf]

SI1: Sampling dates and sample overview

Sampling event, date and number of sexed pellets collected at Chiemsee (Bavaria, Germany) cormorant colony. Additionally, the number of fish species with measurable hard parts and the number of molecularly detected fish species are displayed for each sampling date with the sex-specific numbers in parenthesis: "(female / male)".

no.	sampling date	sexed pellets (female / male)	fish species with measurable hard parts: all pellets (female / male)	molecularly detected fish species: all pellets (female / male)
1	30th March 2012	1 (1 / 0)	0 (0 / -)	0 (0 / -)
2	12th April 2012	43 (26/ 17)	12 (9 / 8)	17 (13 / 12)
3	27th April 2012	21 (12 / 9)	7 (6 / 2)	9 (8 / 6)
4	10th May 2012	14 (4 / 10)	5 (2 / 4)	8 (4 / 6)
5	25th May 2012	19 (3 / 16)	5 (3 / 5)	11 (7 / 8)
6	6th June 2012	24 (5 / 19)	8 (3 / 7)	15 (5 / 13)
7	21st June 2012	12 (2 / 10)	7 (1 / 6)	10 (0 / 10)
8	5th July 2012	11 (3 / 8)	7 (3 / 6)	10 (3 / 9)
9	18th July 2012	13 (3 / 10)	3 (1 / 2)	9 (3 / 8)
10	25th March 2013	7 (4 / 3)	2 (1 / 1)	8 (5 / 5)
11	9th April 2013	11 (7 / 4)	3 (3 / 1)	12 (11 / 2)
12	25th April 2013	10 (3 / 7)	6 (2 / 5)	12 (11 / 7)
13	9th May 2013	23 (10 / 13)	7 (3 / 7)	9 (4 / 9)
14	25th May 2013	23 (14 / 9)	4 (4 / 2)	9 (6 / 6)
15	25th June 2013	58 (27 / 31)	5 (4 / 5)	18 (12 / 17)
16	5th July 2013	82 (40 / 42)	7 (5 / 6)	17 (14 / 15)
17	17th July 2013	15 (8 / 7)	4 (4 / 1)	6 (5 / 4)
18	31st July 2013	16 (8 / 8)	3 (2 / 1)	8 (7 / 5)
19	13th August 2013	12 (7 / 5)	5 (4 / 3)	13 (9 / 6)
