# Supplementary material for: Sex‐specific prey partitioning in breeding piscivorous birds examined via a novel, noninvasive approach
Source: Ecol Evol. 2018 Aug 14;8(17):8985–98. doi: 10.1002/ece3.4421 (PMC6157673; doi:10.1002/ece3.4421)
Supplement: Supplementary file 2 [file ECE3-8-8985-s002.pdf]

SI 2: Cormorant reference samples, sexing PCR, and electrophoretic visualisation

Tissue samples and pellets obtained from cormorants of known sex

Cormorant muscle tissue was obtained from 35 shot cormorants (cf. Oehm, Thalinger, Mayr & Traugott 2016) whose sex was identified through dissection. Small pieces of muscle tissue were removed with DNA-free tools and stored in ethanol at –32 °C upon DNA extraction. Muscle tissue was dried to remove ethanol before adding 200 µl of lysis buffer consisting of: TES-buffer (0.1 M TRIS, 10 mM EDTA, 2% sodium dodecyl sulphate; pH 8) and Proteinase K (20 mg/ml) in a ratio of 190 : 1. Incubation and DNA extraction was carried out as described in the presented methods and in Thalinger, Oehm, Mayr, Obwexer, Zeisler and Traugott (2016). Cormorant pellets (n = 38) produced by birds of known sex were obtained during a one month feeding trial on captive cormorants at "Wilhelma The Zoological and Botanical Gardens" (Stuttgart, Germany) (Thalinger, Oehm, Obwexer & Traugott 2017) and processed likewise.

PCR optimization

The primers 2550F and 2718R, targeting the chromodomain-helicase-DNA-binding protein 1 (CHD 1) gene (Fridolfsson & Ellegren 1999), result in a single amplicon (652 bp) for male cormorants and two amplicons (459 bp and 652 bp) for female cormorants. PCR conditions were optimized with regard to PCR chemistry and cycling conditions. The above described cormorant muscle tissue extracts and pellets of known origin were used to check for correct target DNA amplification. Furthermore, fish muscle tissue extracts (cf. Thalinger *et al.* 2016), were utilized to rule out false amplification based on fish DNA.

PCR conditions and capillary electrophoresis

For the final 10 µl PCRs the Multiplex PCR Kit (QIAGEN) was used including 1.5 µl of DNA extract, 1 × reaction mix, 1 mM forward and reverse primer respectively, 5 µg BSA and PCR-grade water to adjust the volume. The following thermocycling conditions were found most suitable for amplification i.e. all pellets produced by captive birds of known sex were correctly sexed and no fish DNA was amplified: 15 min at 95 °C, 35 cycles of 30 s at 94 °C, 90 s at 55 °C, 1 min at 70 °C, and 10 min at 72 °C once.

The automatic capillary electrophoresis system QIAxcel and the QIAxcel Biocalculator software, version 3.2 (Method AL320, QIAGEN) were used for PCR product separation and analysis. The detection threshold was set at 0.1 relative fluorescence units (RFUs) for the samples displaying both target bands (i.e. females) and at 0.3 RFUs for samples displaying only a single band (i.e. males) to correct for unequal amplification success (Fig. A below).

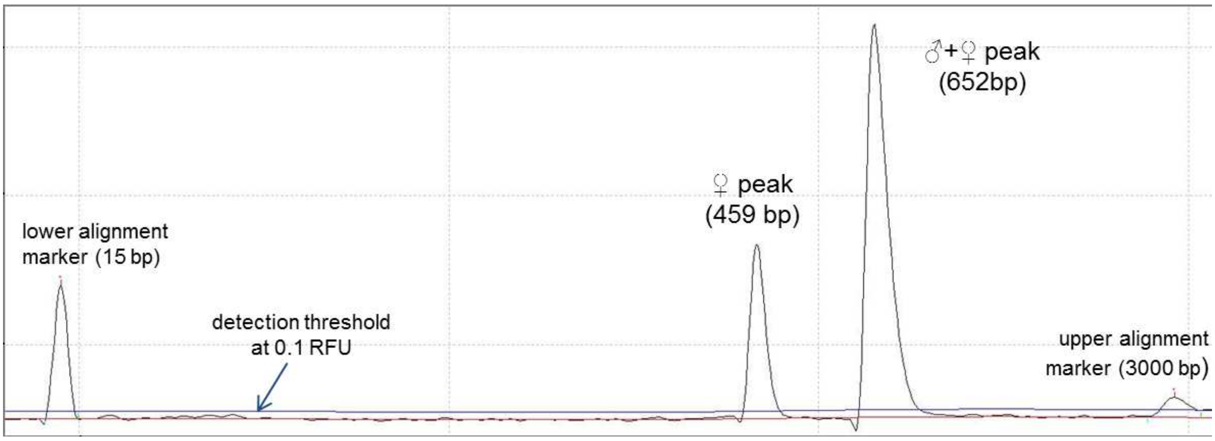

Figure A: QIAxcel image of DNA amplified from a cormorant pellet produced by a female bird. The x-axis provides the passing time during capillary electrophoresis where shorter DNA amplicons pass the measuring point earlier than longer ones. The y-axis displays the relative fluorescence units (RFUs) i.e. signal proxy of the number of amplicons generated. The lower and upper alignment marker (15 bp and 3 000 bp) are used to align several samples. The female peak (459 bp) and the male / female peak (652 bp) show the distinct difference in peak height.

References

Fridolfsson AK, Ellegren H (1999) A simple and universal method for molecular sexing of non-ratite birds. *Journal of Avian Biology* **30**, 116-121.

Oehm J, Thalinger B, Mayr H, Traugott M (2016) Maximizing dietary information retrievable from carcasses of Great Cormorants *Phalacrocorax carbo* using a combined morphological and molecular analytical approach. *Ibis* **158**, 51–60.

Thalinger B, Oehm J, Mayr H, Obwexer A, Zeisler C, Traugott M (2016) Molecular prey identification in Central European piscivores. *Molecular Ecology Resources* **16**, 123-137.

Thalinger B, Oehm J, Obwexer A, Traugott M (2017) The influence of meal size on prey DNA detectability in piscivorous birds. *Molecular Ecology Resources* **17**, e174-e186.
