# Supplementary material for: Sex‐specific prey partitioning in breeding piscivorous birds examined via a novel, noninvasive approach
Source: Ecol Evol. 2018 Aug 14;8(17):8985–98. doi: 10.1002/ece3.4421 (PMC6157673; doi:10.1002/ece3.4421)
Supplement: Supplementary file 3 [file ECE3-8-8985-s003.pdf]

SI 3: Explanation of sequence alignment

Sequence alignment with male (CHD1Z) and female (CHD1W) sequences of the chromodomain-helicase-DNA-binding protein 1 (CHD1) in 5' – 3' direction, including reference sequences from GenBank (two for CHD1Z and CHD1W each). The generated sequences were obtained from extracts of either muscle tissue of shot cormorants that were identified morphologically prior to the molecular analysis (K-samples) or regurgitate pellet samples that were collected during the sampling season in 2012 (between 29th March and 10th May) at the shore of Chiemsee (S-samples). There are slight differences in the CHD1Z sequences of male cormorants. At about 400bp in the alignment a triplet of base pairs (AAG) is missing. For both variants a reference sequence has been found in GenBank. The variation or more precisely the loss of one triplet in our samples only occurs in the tissue extracts, which were taken from individuals that did not belong to the colony at Chiemsee.
