# Supplementary material for: Sex‐specific prey partitioning in breeding piscivorous birds examined via a novel, noninvasive approach
Source: Ecol Evol. 2018 Aug 14;8(17):8985–98. doi: 10.1002/ece3.4421 (PMC6157673; doi:10.1002/ece3.4421)
Supplement: Supplementary file 4 [file ECE3-8-8985-s004.pdf]

SI 4 fish-length regression formulae

Regression formulae for the calculation of fish lengths; columns denote: fish species (common name and Latin name), regression equations for one or more bony structures, unit of the calculated fish lengh, R² marking the fit of the respective regression equation to the reference data, number of fish individuals used for regression calculations (n), the water body of origin of the fish or the Literature cource of the formulae, and the minimum and maximum size of the fish included in the calculations. The following abbreviations are used: TL: total fish length [mm], Ot: length of sagittal otolith [mm], Pha: width of pharyngeal bone [mm], Den: length of dentale [mm], ChP: length of chewing pad [mm], Po: length of preopercular [mm].

Fish species	Regression equation	Fish length unit	R^2	n	Water Body / Literature	min. size [cm]	max. size [cm]
Eel (*Anguilla anguilla*)	TL = 109.25*Ot + 204.13	mm	0.4273	6	Alz	53	66
Barbel (*Barbus barbus*)	TL = 10.667*Pha + 91.667	mm	0.7695	3	Alz	16.8	36
Whitefish (*Coregonus spp.*)	TL = 43.153*Ot + 13.151	mm	0.9396	18	Chiemsee, Hartsee, Langenbürgener See	23.8	37
Pike (*Esox lucius*)	TL = 44.545*Ot + 88.727<br>TL = 0.498*Den + 23.701	mm	0.0760<br>0.3026	6<br>4	Alz, Überseer Bach	19	36.2
Pike (*Esox lucius*)	TL = 7.285*Ot – 7.637<br>TL = 0.698*Den+3.913	cm	0.91<br>0.9877	18<br>7	Cech, Cech, Kubecka, Prchalova and Drastik (2008)	24.5	41
Dace (*Leuciscus leuciscus*)	TL = 18.006*ChP + 105.17<br>TL = 16.556*Pha + 7.333	mm	0.7163<br>0.6463	5<br>4	Überseer Bach	14	21.1
Burbot (*Lota lota*)	TL = 52.621*Ot - 86.164	mm	0.9648	5	Salzach	13.2	31
Rainbow trout (*Oncorhynchus mykiss*)	TL = 91.475*Ot – 66.68<br>TL = 13.069*Den + 32.46	mm	0.9521<br>0.9811	8	Inn, Tiroler Achen	12.5	39
Perch (*Perca fluviatilis*)	TL = 24.952*Ot + 16.154	mm	0.9114	52	Chiemsee, Abtsee, Simssee, Langbürgener See, Hartsee, Weitsee, Alz, Altwasser Osterbuchberg, Almfischerweiher	6.4	24
Perch (*Perca fluviatilis*)	TL = 0.882*Po + 0.556	cm	0.98	47	Emmrich and Düttmann (2011)	5.7	27.1
Roach (*Rutilus rutilus*)	TL = 15.907*ChP + 69.636<br>TL = 11.277*Pha + 33.261	mm	0.9245<br>0.9245	23<br>31	Chiemsee, Abtsee, Hartsee, Langenbürgener See, Weitsee, Baggerweiher Seethal	7.8	30

Species	Regression equation	Fish length unit	R^2	n	Water Body / Literature	min. size [cm]	max. size [cm]
Brown trout (*Salmo trutta*)	TL = 65.245*Ot - 18.64<br>TL = 7.171*Den + 88.33	mm	0.8501<br>0.7474	12<br>12	Weißach, Überseer Bach, Traun	11.5	38
Rudd (*Scardinius erythrophthalmus*)	TL = 18.934*ChP + 57.229<br>TL = 12.253*Pha + 19.826	mm	0.8657<br>0.8063	13<br>15	Hartsee, Langenbürgener See, Weitsee, Baggerweiher Seethal	7	16.1
Grayling (*Thymallus thymallus*)	TL = 85.376*Ot - 70.917	mm	0.7606	5	Salzach, Inn	28.5	52
Tench (*Tinca tinca*)	TL = 29.323*ChP + 31.417<br>TL = 12.643*Pha - 1.9864	mm	0.9815<br>0.9447	5<br>6	Altwasser Osterbuchberg, Baggerweiher Seethal	10.7	29
Pikeperch (*Sander lucioperca*)	TL = 4.688*Ot - 4.6704	cm	0.93	59	Emmrich and Düttmann (2011)	10.4	38.3
Ruffe (*Gymnocephalus cernua*)	TL = 2.27*Ot - 0.0459	cm	0.97	64	Emmrich and Düttmann (2011)	5,4	18,5
Common carp (*Cyprinus carpio*)	TL = 1.110*Pha - 2.8219<br>TL = (0.92*ChP*3.28 - 2.93)*1.15	cm	0.9854	13	Cech *et al.* (2008)<br>Gwiazda (2004)	6.4	53
Bream (*Abramis brama*)	TL = 1.695*Pha + 0.1201<br>TL = 41.805*ChP + 7.826	cm	0.99	27	Emmrich and Düttmann (2011)<br>Gwiazda (2004)	6.8	30.1
Grass carp (*Ctenopharyngodon idella*)	TL = 1.693*Pha - 15.2	cm	0.9992	3	Cech *et al.* (2008)	48.0	73.5

References

Cech M, Cech P, Kubecka J, Prchalova M, Drastik V (2008) Size Selectivity in Summer and Winter Diets of Great Cormorant (*Phalacrocorax carbo*): Does it Reflect Season-Dependent Difference in Foraging Efficiency? *Waterbirds* **31**, 438-447.

Emmrich M, Düttmann H (2011) Seasonal shifts in diet composition of Great Cormorants *Phalacrocorax carbo sinensis* foraging at a shallow eutrophic inland lake. *Ardea* **99**, 207-216.

Gwiazda R (2004) Fish in the diet of the Cormorant and the Yellow-legged Gull breeding near fish ponds (upper Vistula river valley, southern Poland) – preliminary study. *Acta zoologica cracoviensia* **47**, 17-26.
